# Preliminary Results of a Structural Health Monitoring System Application for Real-Time Debonding Detection on a Full-Scale Composite Spar

**DOI:** 10.3390/s23010455

**Published:** 2023-01-01

**Authors:** Monica Ciminello, Bogdan Sikorski, Bernardino Galasso, Lorenzo Pellone, Umberto Mercurio, Antonio Concilio, Gianvito Apuleo, Aniello Cozzolino, Iddo Kressel, Shay Shoham, Moshe Tur

**Affiliations:** 1 Adaptive Structures Division, The Italian Aerospace Research Centre (CIRA), 81043 Capua, Italy; 2 Research Division, Piaggio Aerospace Industries, 81043 Capua, Italy; 3 Advanced Structural Technologies, Engineering Center, Israel Aerospace Industries (IAI), Ben Gurion International Airport, Tel Aviv 70100, Israel; 4 School of Electrical Engineering, Tel-Aviv University (TAU), Tel Aviv 70100, Israel

**Keywords:** structural health monitoring, real-time processing, composite structures, sensors, damage characterization, smart devices

## Abstract

The present paper reports the outcomes of activities concerning a real-time SHM system for debonding flaw detection based on ground testing of an aircraft structural component as a basis for condition-based maintenance. In this application, a damage detection method unrelated to structural or load models is investigated. In the reported application, the system is applied for real-time detection of two flaws, kissing bond type, artificially deployed over a full-scale composite spar under the action of external bending loads. The proposed algorithm, local high-edge onset (LHEO), detects damage as an edge onset in both the space and time domains, correlating current strain levels to next strain levels within a sliding inner product proportional to the sensor step and the acquisition time interval, respectively. Real-time implementation can run on a consumer-grade computer. The SHM algorithm was written in Matlab and compiled as a Python module, then called from a multiprocess wrapper code with separate operations for data reception and data elaboration. The proposed SHM system is made of FBG arrays, an interrogator, an in-house SHM code, an original decoding software (SW) for real-time implementation of multiple SHM algorithms and a continuous interface with an external operator.

## 1. Introduction

In recent years, in order to ensure safe operation of UAVs, especially over populated areas, the industry has moved towards establishing airworthiness requirements, such as STANAG 4671 [1], moving away from commercial manned aircraft airworthiness regulations. An attractive option for maintenance of structural integrity is the use of structural health monitoring (SHM) systems. The aim of SHM is the autonomous structural airworthy assessment of individual vehicles, alerting for maintenance actions only as needed. It is expected that when fully developed, SHM will qualify as one of the ‘repeatable and reliable non-destructive inspection techniques’ mentioned in the aforementioned regulations.

This general case has a specific problem when it comes to bonded structures. Bonding can generate significant advantages for the production of composites, which can be reduced by the applicable directives. STANAG 4671 states the criteria that shall be followed in designing a bonded structure in the USAR.573(a)(5) based on the same criteria present in the EASA and FAA commercial manned regulations. The cited standard states that if the failure of a bonded joint would result in the catastrophic loss of the entire aircraft, it is necessary to demonstrate that the design is compliant through at least one of the following criteria:

- Max debonding of joints should be compliant with load-bearing capability and shall be verified numerically and analytically, or test; debonding exceeding those sizes shall be avoided by design;
- Experiments shall be conducted on each single manufactured component/ subsystem undergoing established critical loads, applied to any endangered mechanical link;
- Verified and trustworthy Non-Destructive Inspection (NDI) shall be defined, ensuring the robustness of each mechanical link.

Of the indicated criteria, the first is generally referred to by aircraft designers; the usual solution consists of adding additional (chicken) fasteners in order to prevent the growth of disbonding beyond the size compatible with the limit loads. They are actually implemented but cause relevant penalties in terms of weight and installation costs. It should be also considered that drilling composites produces further risks of damage onset; greater thicknesses are therefore required, adding further weight. For instance, on some aerodynamic parts, the use of flared-head fasteners that do not affect the continuity of the surface is required. The regulation prescribes that the thickness of the related flared part should not exceed 2/3 of the total thickness. Therefore, once the fastener has been selected, the minimum thickness of the plate is defined. In many cases, such as wing tip sections, the resulting thickness is well beyond that required to withstand loads. Suitable bonding technology could allow for significant weight savings. Repair patches are another example of bonded solutions. If pure adhesive solutions are used without mechanical joints, authorities request demonstration that the structure is able to withstand limit loads increased by an additional safety factor generally set to 1.2 without concurrency of possible repairs. Adoption of bonding for primary structures entails serious consequences for certification, reducing the viability of those options.

The second criterion is almost never adopted because, owing to impractical serial production costs. It would require demonstration that each product is able to withstand limit loads; this would mean testing each manufactured component (wings) at those force levels. However, it would not be even sufficient according to AMC20-29, which requires independent experimental verification of the time resistance vs. environmental degradation and fatigue phenomena of glued joints.

The third criterion represents an interesting solution, as it does not require additional mechanical fasteners—only the assessment of the quality of glued joints by NDI. Even ultrasonic techniques, which are widely used and accepted as a reference tool to check for the presence of defects in a composite structure, are not able to individuate bonding layer discontinuity (missing adhesive) or mechanical strength ineffectiveness (for chemical problems or other kinds of adhesive degradation). However, the same EASA AMC20-29 [2] indicates this third method as that which is most open to future developments and progress of NDI technologies, indicating SHM as a promising strategy.

It is expected that when fully developed, SHM will qualify as one of the ‘repeatable and reliable non-destructive inspection techniques’ stipulated in the aforementioned regulations, allowing for the application of the third criteria rather than the first one, providing more efficient bonding of the composite primary structure. In the following section, an application in this field is presented, considering aspect of “real-time monitoring” of particularly critical bonding lines, such as those between the spar and skin panels of a wing box.

The concept of SHM has been discussed for many years. Among the available SHM technologies, the use of fiber optics sensing and, in particular, the use of multiplexed fiber Bragg gratings (FBGs) appears quite attractive. A variety of sensors is required to provide critical information about the structural health during fabrication, testing and service lifetime. Fiber optic sensors (FOS) are considered for aerospace applications, owing to their many advantages that can lead to solutions with the potential to outperform their conventional counterparts. Fiber optic sensors are quite flexible and tolerant to environmental conditions and electromagnetic interferences. In addition, their small diameter allows for easy embedding within large composite-material-based structural components, such as many UAV wings [3]. The ability to monitor both composite and metal structures by optical sensors has already been successfully verified [4]. Examples of in-flight applications can be found in literature, even by surface bonded sensors [5]. The application of FBG sensors for UAV leading edges for bird strike damage assessment was tested on the ground [6,7]. In-flight measurements using surface-bonded FBG sensors were successfully applied for UAV wing shape sensing, [8,9]. Instead of point sensors like FBG, embedded optical distributed strain sensing was tested on the ground for damage detection purposes on aeronautical structures [8].

Fiber Bragg Grating (FBG) sensors have been successfully applied to a pressure vessel under loading conditions and a curved fuselage panel. In that case, by applying multivariable statistical analysis (i.e., principal component analysis), it was possible to reduce the complexity and size of the samples, revealing patterns and trends that could have been hidden under the data. Given the stochastic nature of the measures, the computation of the sample average and standard deviation of the baseline and current (possibly damaged) strain measurements is another possible solution [10]. Computation of metrics was performed, producing a damage alert when the value exceeded a predefined limit. Further signal processing methodologies for SHM can be retrieved from the bibliography of the above-cited study. Most of them use the differences between a baseline set of measurements and the dataset in an unknown structural state. This unknown state is somehow correlated to the reference state according to different approaches and different damage index (DI) indicators. Other techniques take advantage of the strain difference revealed by two close sensors to detect the possible presence of flaws [11,12,13,14,15], irrespective of the static or dynamic nature of the excitation. Damage alters the stiffness and strain field distribution within the structure, even if in a very limited surrounding area.

Based on these experiences, the procedure proposed herein is a non-model damage identification method based on deviation features extracted from the current “referenced” strain profile of a fiber optic sensor array. The core principle of the algorithm is to correlate structural damage with maximum local gradient variations as the edge detection technique typically used in digital image processing applied to a 1D vector as a cost-effective and time-saving procedure.

## 2. Aims and Motivations of the Research

The main aim of the activity presented herein is to demonstrate the functionality of a real-time SHM system targeting the detection of damage on a generic structural element. Herein, the referred test article is a composite beam with a complex design.

The proposed system is based on a real-time module, allowing for management of data provided by a four-channel interrogator and redistribution of this information to different SHM algorithms, with the aim of detecting the possible presence of flaws. In the described setup, SHM algorithms developed by CIRA (Italian Aerospace Research Centre) and IAI/TAU (Israel Aerospace Industries/Tel-Aviv University) were simultaneously deployed, operating concurrently on the same data. The generated output is managed by a real-time module, which transforms the results into sound (or even visual) cues to attract the attention of a generic user. In this regard, the measure of the actual real-time system considered herein is the difference between the instants correlated to two consecutive outputs; such a time interval was evaluated to typically be approximately one second.

The specific architecture tested herein comprises two different FBG arrays for strain measurement placed on different pre-existing flaws of the beam. The structure is excited by a quasi-static force generated by a 10 kN electromechanical actuator. During the demonstration, all system components are implemented, and the external load is representative of the operational load. The experimental setup permits evaluation of the minimum strain level threshold as the SHM system starts, providing reliable data (i.e., not a single, temporarily isolated event but a recursive indication of detection), resulting in approximately hundreds of microstrains, which is roughly equivalent to 10% of the max load applied in the referred test and therefore in line with the expressed needs. In summary, the aims of this activity are:

- Verifying the real-time functionality of the proposed real-time SHM system;
- Verifying the capability of the developed SHM algorithms;
- Verifying the capability of the system to simultaneously handle several SHM algorithms; and
- Quantifying the expected strain detection threshold.

## 3. The SHM System

Most robust SHM systems are reference-driven; damage recognition is based on an accurate model of the healthy structure. Then, analysis and formalization of decision-making rules are provided on the basis of expertise. Nevertheless, in some application scenarios, real loading conditions are not always simple to reproduce and to predict; therefore, detection methods with unknown input force could be more desirable despite a loss of accuracy. The idea proposed herein is to detect damage occurrences without any preliminary characterization of the healthy component, instead using measured strain value [16,17,18,19,20]. To this end, the effect of the damage over the strain signature is exploited in order to identify a set of features that allows for an effective representation of the present faults. Core diagnosis is implemented using the cross-correlation function. This is a standard method for estimating the correlation level between two signal and is intended as a measure of similarity of the two signals.

The real-time system is based on the choice of Ethernet as the communication bus. Networking provides the necessary integration between various subsystems. Individual modules comprise the whole system can run on separate dedicated computers or on the same personal computer (PC), provided it has sufficient characteristics (mass memory, CPU speed, etc.).

### 3.1. Damage Detection Algorithm

**Figure 1:**
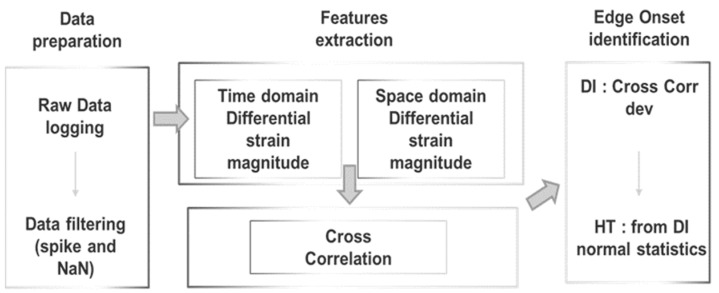
Flow chart of the LHEO algorithm. DI, damage index; HT, high threshold level.

The LHEO (local high-edge onset) algorithm developed by CIRA considers structural damage as an edge discontinuity along the strain energy signature. Its logic diagram is reported in Figure 1. The onset of edge signals can be tracked in both the space and time domains correlating the measured rate of similarity of current strain values to next strain values using a sliding inner product as a function of the sensor step (minimum lag of two consecutive sensors at a certain instant) and as a function of time step (minimum lag of two consecutive time acquisitions at a sensor position). The “sliding lag” dimension must be compliant with the sensor system, i.e., the measuring point density and distribution, which is a design spec according to the damage dimension to be detected.

For the sake of clarity, the cross-correlation function [18,19,20] represents the measure of similarity of two signals as a function of a time shift or a spatial translation applied to one of them.

(1) $$R_{ij}\left(t\right)=\frac{1}{N}\overset{N-1}{\underset{l=0}{\sum }}x_{i}\left(t\right)x_{j}\left(t+\tau \right)$$

where N is the number of structural responses in the sample, *τ* is the time delay; when *i* = *j*, Equation (1) is the autocorrelation function. Considering two real signals (*i*-th and *j*-th) and a general *x*-axis (whatever domain *x* represents), the cross correlation can be calculated to determine the extent to which the *i*-th signal must be anticipated along x-axis to make it identical to the reference *j*-th signal. The formula essentially anticipates the signal along the axis, calculating the integral of the product for each possible value of the displacement. Assuming that the structural damage is in the form of a change in the structural stiffness, the stiffness value at position *i* of the damaged structure can then be expressed as:

(2) $$K_{i}^{d}=\theta_{i}K_{i}$$

where $K_{i}$ is the stiffness of the *i-th* element in the reference state (undamaged, baseline or whatever reference status is adopted); and $\theta_{i}$ is defined as the stiffness fraction relative to the reference stiffness of the *i*-th element; and θ = 0 denotes that the element loses its stiffness completely, whereas θ = 1 indicates that the element remains intact.

For a generic structural response under load f, the equation for an N-degrees-of-freedom (N-DOFs) viscous damped structure is expressed as:

(3) $$f\left(t\right)=M\ddot{x}\left(t\right)+C\;\dot{x}\left(t\right)+Kx\left(t\right)$$

where *M*, *C* and *K* are the mass, damping and stiffness matrices, respectively, and $f\left(t\right)$ is the input excitation. For a static or quasistatic condition, it was demonstrated in [19,20,21] that Equation (3) can be written as a function of the strain ($\varepsilon \left(t\right)$):

(4) $$B^{-T}f\left(t\right)=B^{-T}KB^{-1}\varepsilon \left(t\right)$$

where *B* is proportional to the differential operator. By substituting Equation (2) into Equation (4), the response for a damaged structure can be simplified as Equation (5), where subscript *i* refers to the structure element:

(5) $$f_{i}=\theta_{i}K_{i}B^{-1}\varepsilon_{i}$$

or:

(6) $$B\frac{f_{i}}{\theta_{i}K_{i}}=\varepsilon_{i}$$

By considering strain measurements as input signals, the cross-correlation function of Equation (1) can be written using Equation (6) as follows:

(7) $$R_{ij}\left(t\right)=\frac{1}{N}\overset{N-1}{\underset{l=0}{\sum }}\varepsilon_{i}\left(t\right)\varepsilon_{j}\left(t+\Delta \tau \right)$$

The expression of the cross correlation in Equation (7) includes a multiplying constant referring the input force that can be eliminated by normalizing its root mean square value. Two considerations can be made:

1. If the strain at the current acquisition is not affected by any variation with respect to time evolution and, similarly, when the signals at different sensor locations coincide, the value of Equation (7) is maximized and corresponds to autocorrelation.
2. As the goal is to identify a change in the structural stiffness, both strategies (time strain similarity and location strain similarity) can be adopted, and the autocorrelation function can be used as the reference signal for strain energy in the time and space domains (assumed as features).

By setting the upper value of the autocorrelation envelope function of the current responses as a vector:

(8) $$R_{max}\left(t\right)=\left[\max\left(R_{ii}\left(T\right)\right)\right]$$

where i=1,2,…,n is the response from measurement sensor point *i*. The relative change of the cross-correlation function with respect to the reference autocorrelation vector in Equation (8) is defined as a damage index as follows:

(9) $$DI_{i}=\left[\left(R_{ij}\right)]-[R_{max}\right]$$

In the absence of a jump/edge, the damage index (9) is small. On the contrary, if an edge is present, then the two function values in (9) differ considerable. Ultimately, the upper value of the envelope of Equation (9) is used to set the highest limit (HT) for the eligible sensor dataset, whereas the mean value (point value) of this upper envelope is used to set a lower limit (LT). Readouts below LT are discarded, whereas readouts between LT and HT are kept if there is a “link” connecting them and verifying their persistence.

### 3.2. Real-Time System

The starting point for the design of the hardware architecture was the selection of the main hardware element—the interrogator. The data interface type of the interrogator selected for this project, the Aero Mini interrogator from SmartFibres, is Ethernet. Careful analysis revealed that true real-time hardware can be replaced with consumer-grade PCs, along with the use of Ethernet as the principal type of data communication bus. Communication between various system elements occurs via connectionless sockets using the User Datagram Protocol (UDP).

**Figure 2:**
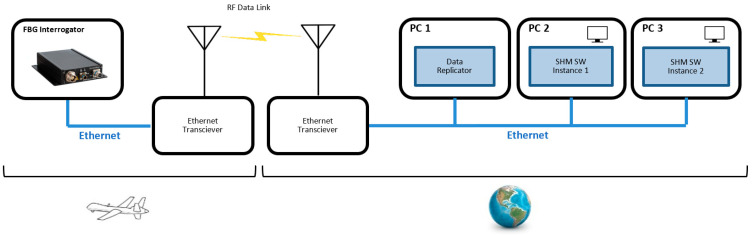
Ethernet as the main communication bus.

Using Ethernet as the principal data communication interface greatly simplifies the complexity of the real-time hardware of the whole data system and also provides flexibility for the execution of the SHM algorithms; each software (SW) module can run on an independent computer or they can run on the same machine. A schematic of the communication architecture is presented in Figure 2.

An FBG interrogator typically interacts with a single application. In order to ensure the possibility of multiple data stream destinations, a data replicator code was created. It is the only code that communicates directly with the interrogator; it configures the interrogator for peak data streaming and sets itself as the destination for the UDP data packets. Each received peak data packet is re-sent to all destinations that process the data. The re-sent data are not modified in any way, thus giving the destination programs the impression that they have been directly communicating with the interrogator. Having multiple SHM processing instances allows for simultaneous elaboration of the real-time data by more than one application. This solution is useful when using multiple SHM algorithms at the same time or when we want to concurrently execute the same code but with different parameters.

The output produced by our executable is an audible tone on the computer’s speakers in case damage is detected. Additionally, some textual data are generated during the program’s execution: the number of data frames processed in each iteration and, in case of detected damage, the indices of the FBGs that report damage.

The SHM algorithm code was originally developed in Matlab. In order to avoid the introduction of potential mathematical errors when using different computational libraries in different programming languages, we opted to continue utilizing Matlab. The SHM algorithm is compiled into a library/module callable from another programming language. This solution has a yet another advantage: it alleviates the need for a Matlab license or any other royalty when executed on the target platform [22]. The compiled module also protects intellectual property, as it is inherently encrypted, thus hiding the source implementation code. Maintaining Matlab as the environment for SHM code development offers complete freedom when continuing the work of refining of the algorithm without the need for repetitive porting of the code into other languages. The rest of code was developed using Python, meaning that the Matlab SHM code is compiled as a Python module.

The Matlab code of the SHM algorithm is written as a callable function, taking a single matrix as input and producing a single vector as output. The input matrix is a collection of a sequence of strain readings from the interrogator. Columns correspond to the strain readings from FBG gratings, and each row corresponds to an instance of time of sampling data. The output vector has the same dimensionality as the number of FBG gratings, and each non-zero value element of the vector corresponds to the FBG with damage detected. The Matlab SHM function is fast and can process thousands of time samples of data at a time; it is incorporated in a Python wrapper program that receives, buffers and processes raw peak data packets from the replicator software (or interrogator) and then calls the Matlab SHM function.

A completely different code was additionally written and utilized with the sole goal of testing and verification of correct handling of more than one data destination by the data replication part of the system. This supplementary code simply presented in real time a graphical plot of the strain values for the individual FBGs. The Python wrapper code was written as multiprocessing to separate the data received from data elaboration and to alleviate execution time restrictions resulting from the Python global interpreter lock (GIL). The data receiving process is a simple loop that waits for data using a blocking *socket.recvfrom*() and then appends the received data into shared memory buffers that are not being used by the data elaboration process. The data elaboration process starts when a sufficient amount of data has been collected in a shared memory buffer. It then instructs the data receiving process to start collecting the arriving data in the other shared memory buffer while it elaborates the currently accumulated data. The elaboration consists mainly of transforming the peak data into strain values, subselection of data and performing data logging if requested.

**Figure 3:**
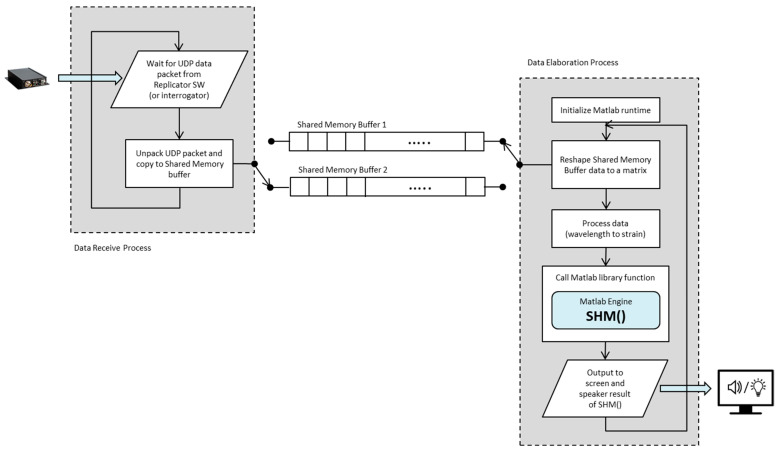
Real-time system flow chart.

If the SHM algorithm detects damage, a signal is sent to a yet another process that provides the audible feedback. A simplified data flow diagram (with the audible feedback process omitted) is presented in Figure 3.

All software components and solutions were selected to keep the choice of operating system open as much as possible. The execution platform can have either Windows, Linux or MacOS installed as the operating system, as all software elements are compatible with all of these platforms. We utilized Windows as the operating system on all computers of the system.

The software implementation of all the elements of the whole system proved to be efficient enough to run on a single high-end computer—in our case, an Intel i7 11800H processor with 32 GB of RAM running up to three instances of different SHM code in real-time without any problems.

## 4. The Test Setup

**Figure 4:**
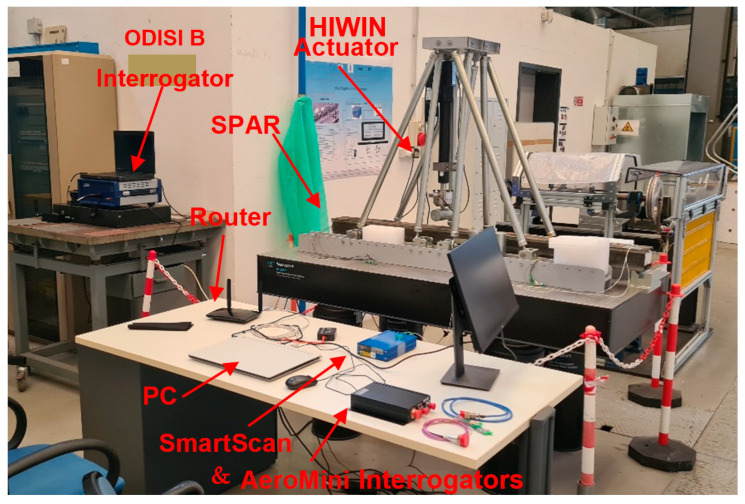
An overall view of the full experimental setup. Details of the subcomponents are provided below.

**Figure 5:**
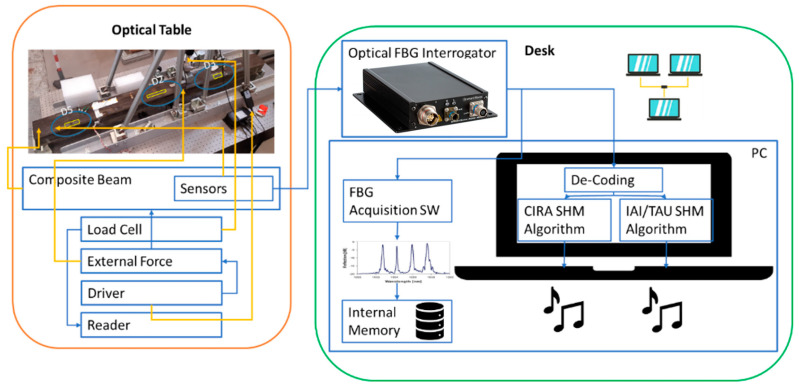
Block diagram of the functionality check of the real-time SHM Code.

**Figure 6:**
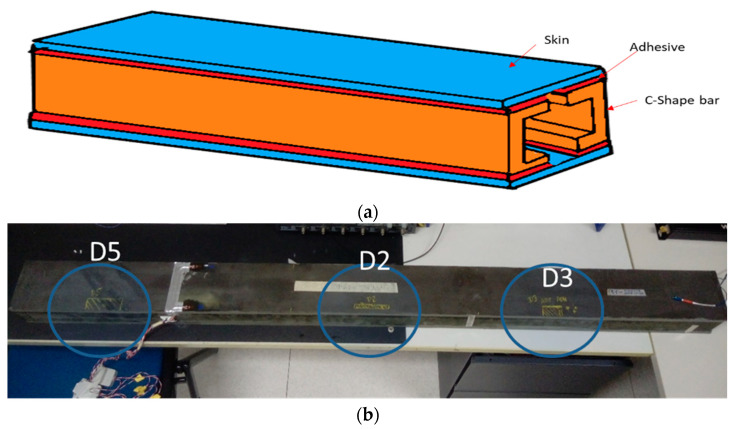
The test article designed and manufactured by IAI: a sketch of the cross-section spar (**a**) and the assembled composite spar (**b**).

To prove the functionality of the system, a dedicated setup was assembled. An overall view of the setup is presented in Figure 4 and Figure 5 (a more detailed view of the spar is presented next in Figure 6).

The test article was installed on a dedicated test rig. The input force was detected by a load cell and visualized by a digital reader. A block diagram of the test is shown in Figure 5, with the aim of verifying the real-time HUMS (health and usage monitoring) code. It uses the composite spar introduced in the next subparagraph.

### 4.1. The Test Article

The test article is a full-scale spar composed of two flat, unidirectional carbon-epoxy tape skins and two C-shaped profile spars bonded to each other using a structural paste adhesive (Figure 6a). The overall dimensions are as follow: length: 1600 mm; width: 120 mm; height: 90 mm.

Three damaged areas were introduced on the upper side of the beam at different positions along the length of the beam (Figure 6b). Damage was applied to the contact between the top caps of the C spars and the plates. In this study, the top flaws are referred to as D5 (70 mm), D2 (80 mm) and D3 (40 mm). The flaws were placed in a symmetric position (D2) and two other non-symmetric positions (D5 and D3) in locations characterized by a constant skin thickness. The only variation for the flaws is the extension length (70 mm for D5 and 40 mm for D3).

### 4.2. The Test Rig

Test execution was planned to verify the algorithm’s ability to detect the location and dimensions of flaws as a function of the distance from the loading point. The strain distribution can change according to load and, in particular, the inlet energy. For this reason, the sensitivity of the system changes accordingly. In this test, a local bending solicitation was preferred to a distributed one, and to accomplish the goal, a dedicated test rig was realized.

**Figure 7:**
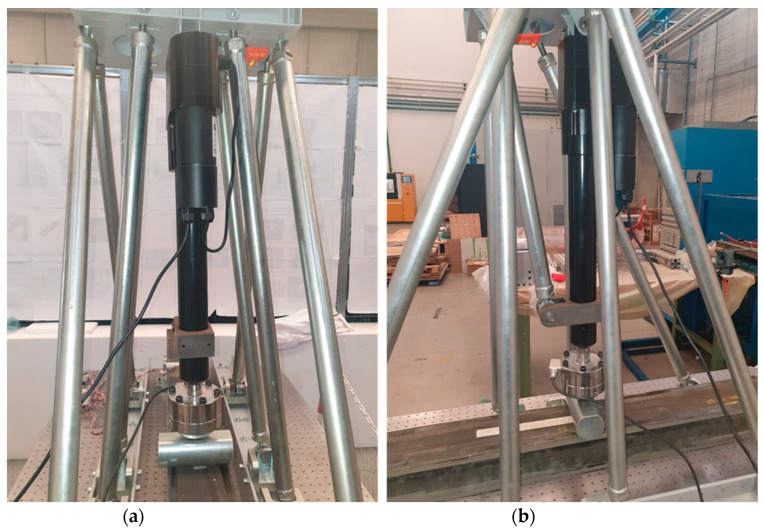
Details of the test rig and linear actuator. Front view (**a**); side view (**b**).

The test rig (Figure 7a,b) was made with steel tie rods and connected to an optical table using two steel profiles. Specifically, the entire test rig was mounted on a Newport production optical table. M6 holes with a 25 mm center distance were drilled in the optical table for fixing parts. The upper ends of the tie rods support a base reinforced with ribs to position a linear actuator. The linear actuator has a rounded moving head to allow for the application of a load of up to 12 kN in a perpendicular manner to the optical table and, in particular, in an incoming direction with respect to the table itself. The actuator was produced by HIWIN, and the model is LAI-2, with a remote controller. The optical table was equipped with a self-levelling system that uses filling tanks located under the work surface, employing pressurized air in order to maintain the actuator on the axis.

Bending tests were conducted on the test article with a load perpendicular to the longitudinal axis of the beam. The beam was slightly raised from the optical table due to the presence of two lateral supports. The support consists of a base that supports a cylinder to reduce local carving effects. The tests involved two different positions of the force with respect to the axis:

1. Symmetrical load: perpendicular force positioned at the center of the beam axis;
2. Asymmetrical load: perpendicular force positioned in the vicinity of one of the supports.

### 4.3. The Sensor Layout

In order to start an experimental correlation study, the following sensor layout was provided according to the damage map and functionality test. In particular, an offline SHM test was performed on damage D3, and an online SHM test was performed on damages D2 and D5.

#### 4.3.1. The Sensor Layout for D3

**Figure 8:**
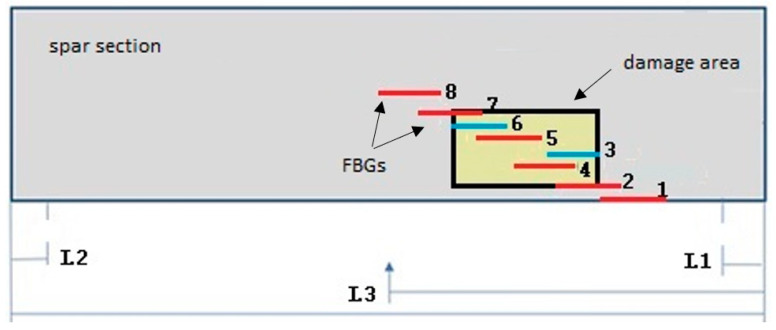
Scheme of the positions of FBGs with respect to beam for the damage area. The FBGs are labelled with progressive numbers from 1 to 8.

Single FBGs were glued in a customized layout. The damaged zone (back edges of the rectangle in Figure 8) was instrumented with 8 sensors partially overlapped in an attempt to provide a quasi continuous strain. This solution is useful to verify the minimum spatial resolution able to detect the damage edge. Figure 8 shows the layout of the position of the FBGs (red and blue segments). L1 and L2 represent the positions of the supports, and L3 represent the position of the force with respect to the length of the beam.

**Figure 9:**
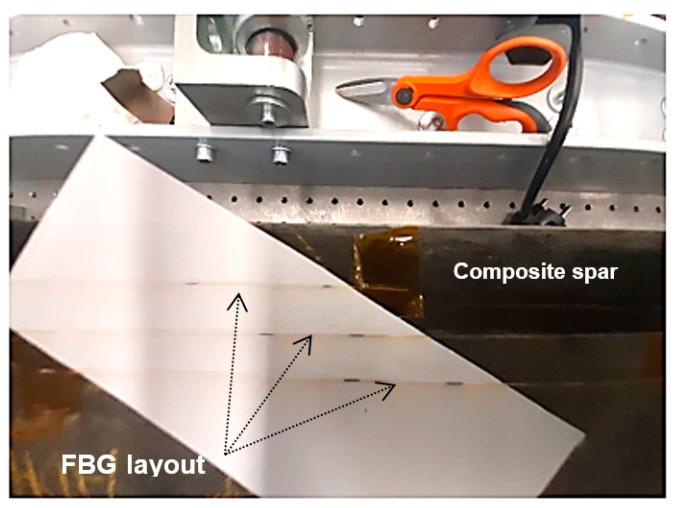
Installation of the FBGs. Layout of three bonded FBGs, each placed on a single fiber.

The blue FBGs (3 and 6) were added to refine the monitored region. Figure 9 shows an example of the partially overlapped FBG layout, in particular for sensors 4, 5 and 7 (Figure 8).

#### 4.3.2. The Sensor Layout for D2 and D5

**Figure 10:**
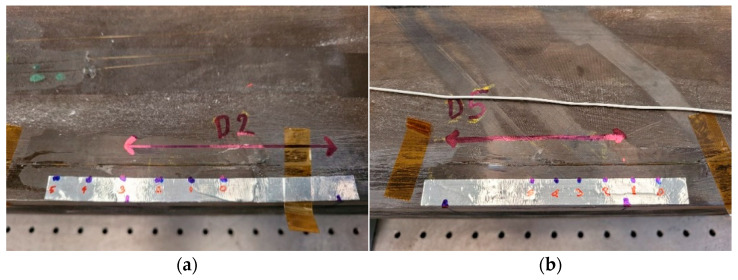
FBG array layout with respect to the damage extension: D2 (**a**); D5 (**b**). Blue dots correspond to the FBG middle point; ID numbers of the sensors from 0 to 6 are indicated in red below the blue dots.

Two 6-FBG arrays were placed along the D2 and D5 flaws with a fixed spatial spacing, each with one of their edges partially covering the healthy and the damaged part of the substrate (Figure 10). The extent of damage is marked by a double arrow. The FBG middle-point position is marked by blue dots. For damage D2, FBGs 2 and 3 are on the edge (Figure 10a); for damage D5, FBGs 1 and 2 are on the edge (Figure 10b). Figure 10 shows the layout of the two arrays.

## 5. Test Results

The functionality of the real-time SHM system was verified for the detection of two different damage locations by running three instances of the code at the same time. Two of these instances involved raw data streaming from two fibers—each one monitoring a single damage area and the third instance running based on data streaming for visual analytics. In the next section, a preliminary numerical analysis of the full-scale spar is presented to provide a strain map signature for damage D3. The outcomes were then used to set specifics for the experimental detection of damages D2 and D5. Details are provided below.

### 5.1. The Numerical Outcome

**Figure 11:**
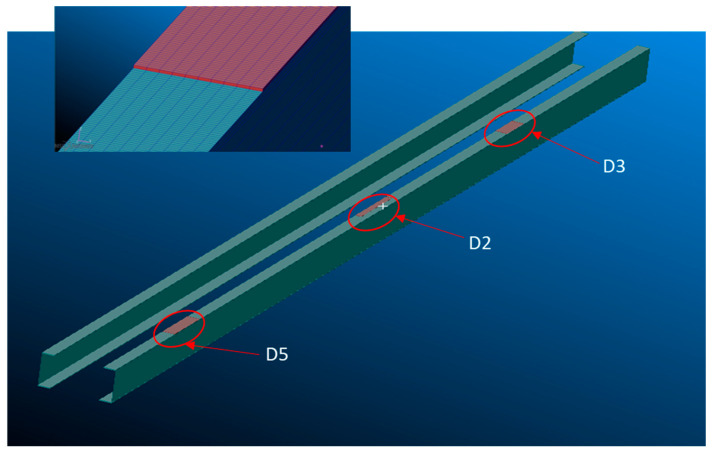
Finite element model of damage.

A finite element (FE) model of the wing spar was realized in order to perform a numerical simulation to tune the SHM algorithm. The FE model represents a typical three-point bending test setup and is constituted by means of shell elements (2D CQUAD) for the skins and spars, whereas 3D elements (CHEXA) with orthotropic properties were used for the paste adhesive. The three artificial damage areas (D2, D3 and D5) were introduced between one cap and the upper skin to simulate the debonded condition by decreasing the material properties of the adhesive (Figure 11).

**Figure 12:**
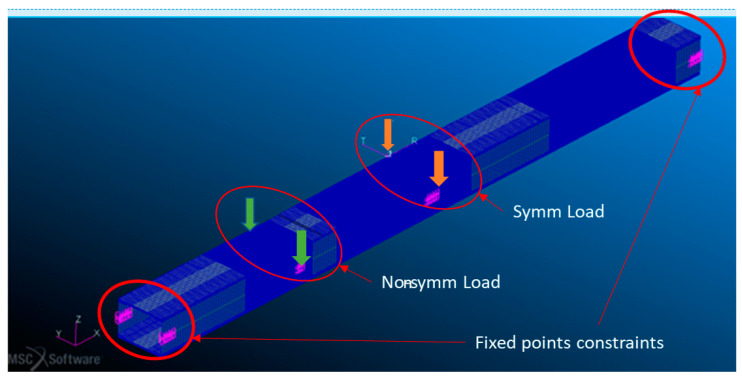
Finite element model of load point.

Multispider elements (RBE2) were applied to introduce the load and constraints. In particular, two multipoint constraints were used to model the fixed supports at the ends of the beam, whereas the load points correspond to the center and ¼ of the length of the beam, respectively, depending on the foreseen test cases (symmetrical and non-symmetrical test case, respectively; Figure 12).

**Figure 13:**
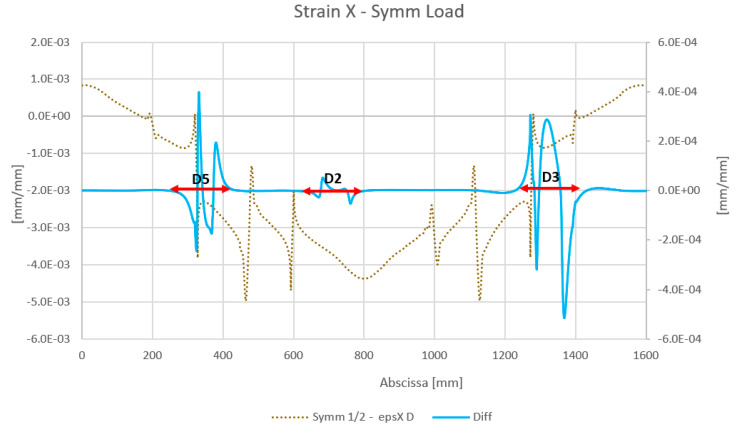
The results of the numerical analysis for the strain map under a symmetric load. The “epsXD” curve refers to the strain (eps) map along the X direction (longitudinal) for the damaged spar. “Diff” refers to the difference in the damaged structure strain signal relative to the undamaged signal.

**Figure 14:**
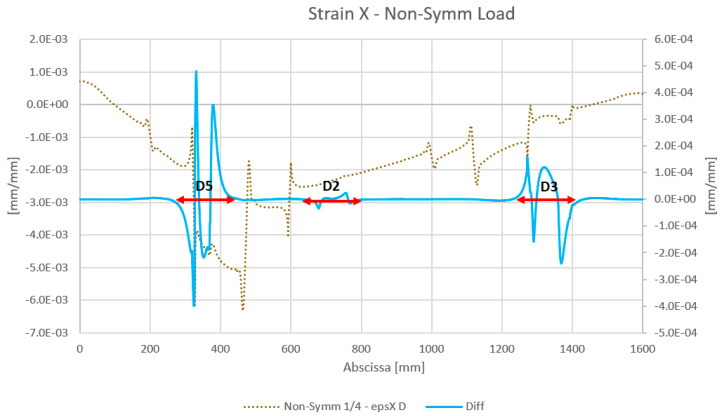
Result of the numerical analysis of the strain map under an asymmetric load. The “epsXD” curve refers to the strain (eps) map along the X direction (longitudinal) for the damaged spar. “Diff” refers to the difference in the damage signal of the damaged structure strain signal relative to the undamaged signal.

The results obtained by the numerical analysis are reported below. The strain values for the reference virtual fiber installed on the wing spar skin for both symmetrical and non-symmetrical load cases are reported, in the diagrams presented in Figure 13 and Figure 14, respectively. In particular, the diagrams show the strain results of the structure with damage. Moreover, in order to obtain a result independent of the initial conditions, a diagram of the difference in the strain values for a damaged and undamaged structure is reported for each case; only debonded zones are highlighted (double arrows).

**Figure 15:**
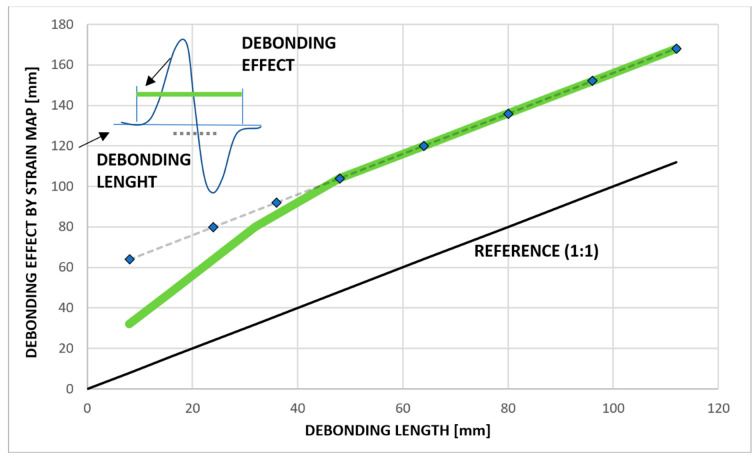
Damage effect over current dimension [18].

The results presented above show that for both test cases, the variation of strains corresponding to debonding zones is restricted to areas close to the damage, whereas the rest of the beam structure does not seem to be influenced by the presence of damage. In addition, the results confirm the effect illustrated in Figure 15 [18]. A strain representation of the debonding region shows that the length over which the structural response is influenced by the presence of a flaw is larger than the flaw itself, depending on the strain energy and damage dimension. The dashed line overlaps the green line, highlighting the linearity of the effect for damage longer than approximately 40 mm; for those values, the effect of the damage exceeds its size by a constant value almost equal to 60 mm. For shorter damage lengths, the law correlating the damage effect to its size is different and tends to approach zero as the flaw vanishes. This means that the SHM algorithm can select sensors within the immediate vicinity of the damage onset. However, this result can be considered valid only for the test article under investigation, and further analyses should be carried out for different structures in order to verify this behavior.

Results reported in Figure 15 show physical phenomenon appearing when moving from the geometrical domain to the deformation domain. These results were obtained considering an FE model mesh of 2.5 mm. This value was chosen to be compliant with the commercially available distributed fiber optic resolution [23] used as reference to define an appropriate FBG array layout to be used for subsequent tests. In particular, we verified that by using such a distributed fiber optic, a damage length of 30 mm was successfully detected. Because the design specs assume a chicken-fastener distance of 80 mm, a lower-density array can be adopted. Nevertheless, it must be considered that from the SHM system point of view, on one hand, the size and density of the sensor array can affect the recognition of a flaw and its effect on strain in terms of effective damage length estimation. On the other hand, SHM algorithm parameters (such as threshold level, sliding inner product lag, etc.) can also affect the recognition of flaws in terms of effective false-positive filtering and effective damage length estimation, taking into considering the oversizing effect observed and reported in Figure 15.

**Figure 16:**
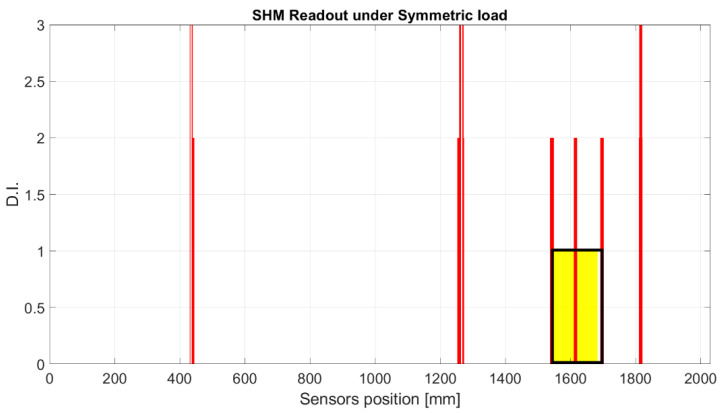
SHM numerical readouts for D3 under a symmetric load. The ordinate refers to the damage index estimated for each spar element. The red bars indicate that the corresponding spar elements provide a local high-strain energy gradient. The red bars close to the yellow rectangle correspond to the edge of the damage, and the other highest bars correspond to structural thickness variations that are known from the design and can be eliminated by offset.

**Figure 17:**
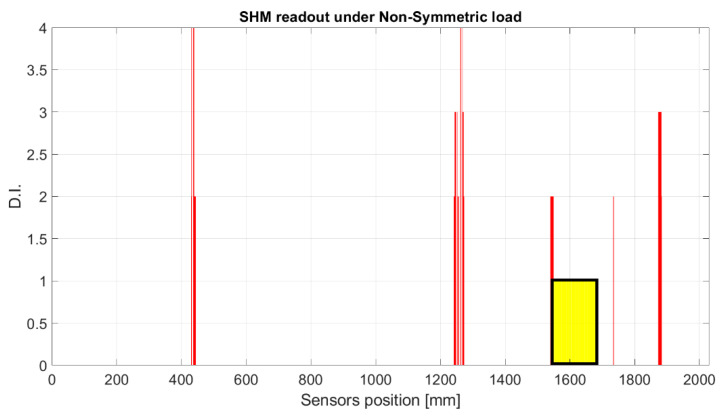
SHM numerical readouts for D3 under an asymmetric load. The ordinate refers to the damage index estimated for each spar element. The red bars indicate that the corresponding spar elements provide a local high-strain energy gradient. The red bars close to the yellow rectangle correspond to the edge of the damage, and the other highest bars correspond to structural thickness variations that are known from the design and can be eliminated by offset.

The strain signal was processed by the SHM system, as shown in the flow chart in Figure 1. The plots provided in Figure 16 and Figure 17 represent the normalized damage index from Equation (9) as a function of the elements of the spar—in this case, with a step mesh length of 2.5 mm. The yellow rectangle with black edges highlights the position of D3, and red lines indicated the postprocessed eligible SHM sensors. The visual readout demonstrates that in both cases, the damage positions and length are well-identified, as the damage edges are marked by the red bars corresponding to the eligible sensors. Nevertheless, some extra outputs are also present. A comparison with the numerical strain map reveals that those sensors actually correspond to a stiffness gradient, owing to the presence of thickness variations along the skin. Those positions are present in both the figures and can possibly be filtered by offset of the initial signature. Furthermore, owing to the different positions of the load point, the strain distribution may change; this is the reason why the level of the damage index is variable, as it depends on the strain energy value of the neighboring sensors processed by the cross-correlation function.

**Figure 18:**
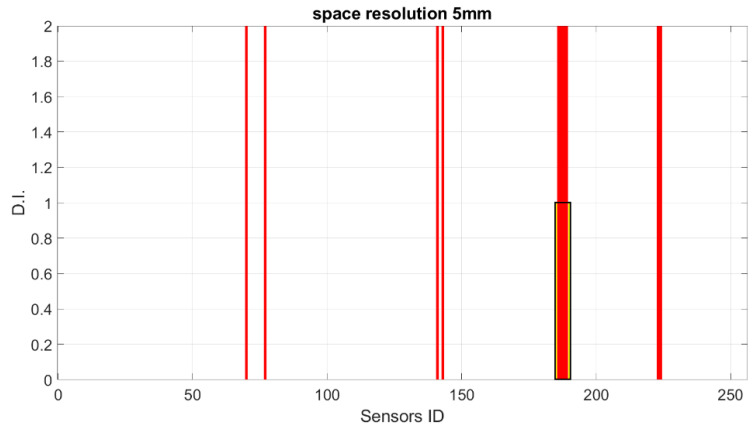
SHM numerical readouts for D3 under a symmetric load with 5 mm resolution. The sensor ID is the number of sensors according to the current spatial resolution.

**Figure 19:**
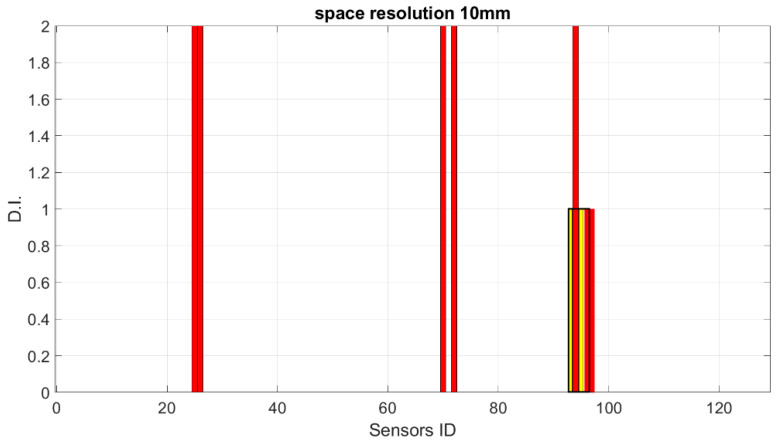
SHM numerical readouts for D3 under a symmetric load with 10 mm resolution. The sensor ID is the number of sensors according to the current spatial resolution.

**Figure 20:**
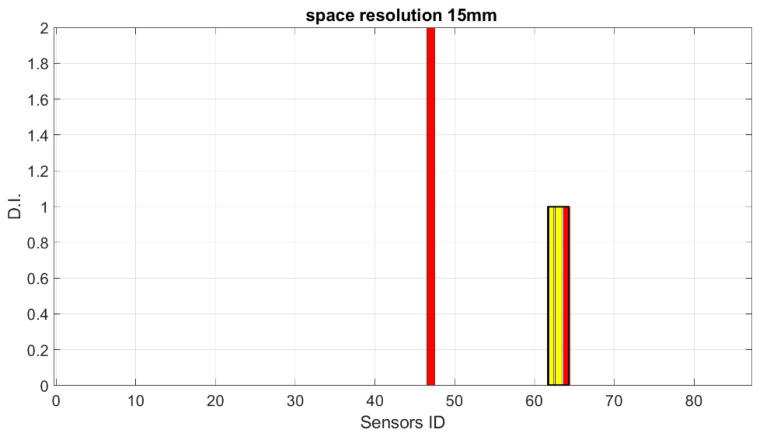
SHM numerical readouts for D3 under a symmetric load with 15 mm resolution. The sensor ID is the number of sensors according to the current spatial resolution.

**Figure 21:**
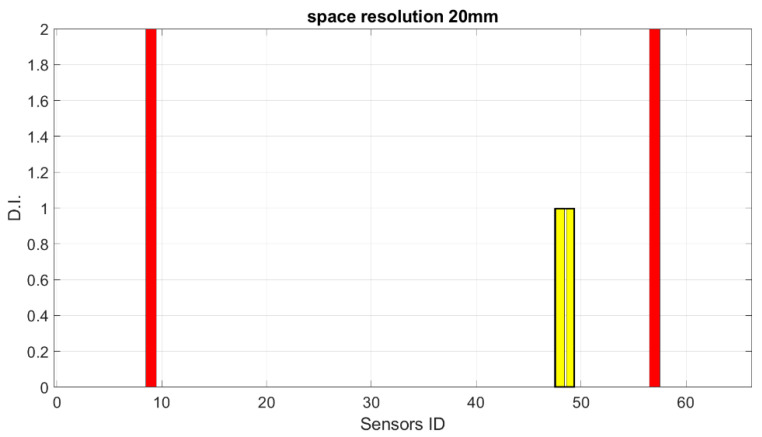
SHM numerical readouts for D3 under a symmetric load with 20 mm resolution. The sensor ID is the number of sensors according to the current spatial resolution.

Starting from these preliminary outcomes, numerical down-sampling is provided, moving from 2.5 mm of the FEM mesh down to 5 mm, 10 mm, 15 mm and 20 mm spatial resolution. In Figure 18, Figure 19, Figure 20 and Figure 21, the damage index is plotted as a function of the sensor elements (with changes in spatial resolution, the ID of total sensors changes accordingly).

The test described above demonstrates that there is a minimum number of sensors that need to be provided to be able to detect damage of a certain length. It is clear that in this case, using a 20 mm sensor step with a 40 mm damage length, the damage detection system fails, as expected according to the Shannon theorem [24].

### 5.2. The Experimental Outcomes

**Figure 22:**
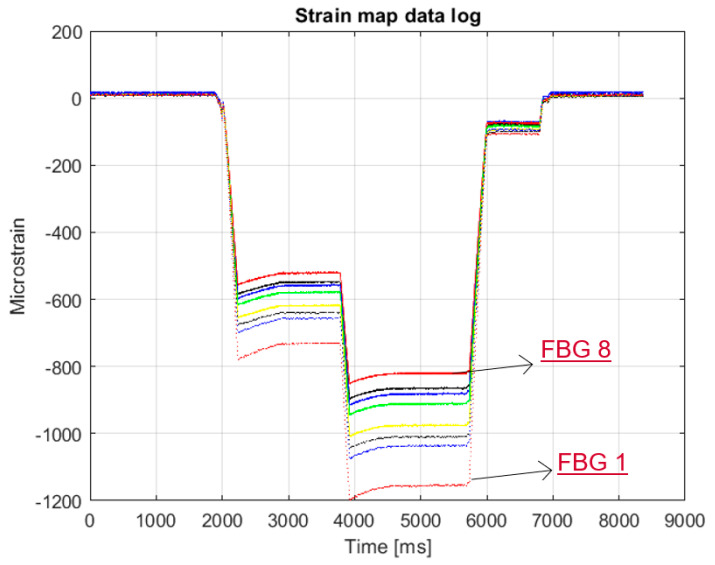
Strain map of eight FBGs monitoring D3 under a quasi-static load. Colors refers to different FBGs starting from the FBG1 to FBG8 in the same order reported in Figure 8.

Starting from the numerical simulations, the first experimental test was used to validate the capability of the system to detect D3 using a 15 mm step array. As many as eight FBGs were surface-bonded (Figure 8). Despite the varied load (Figure 22), the ratios of the strain curves remained constant. This guarantees the stability of the signal of the various FBGs.

**Figure 23:**
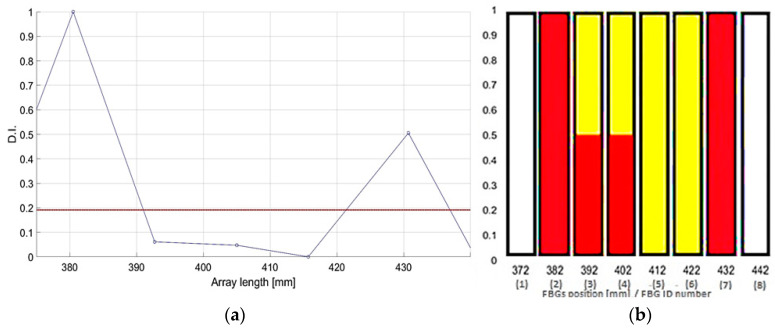
SHM post processing for D3: normalized cross correlation from the FBGs net (**a**); eligible sensor selection indicating the damage edge with highest strain energy gradient (**b**).

The post processing of the input strain provides some sensor selection. In particular, the FBGs are represented by eight bars (Figure 23). The sensors lying within the damage area are colored yellow (from 382 mm to 422 mm). The SHM readout is provided in red. The two peaks exceeding the threshold value in Figure 23b, corresponding to the highest energy gradient, are shown as full red bars.

**Figure 24:**
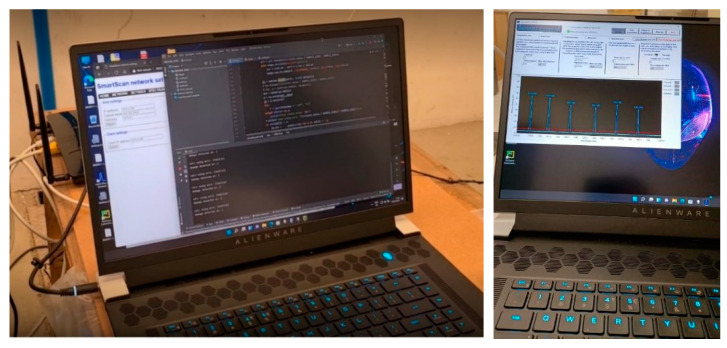
SHM system readout during real-time test.

The next part of the experimental campaign focused on the validation of the real-time system for D2 and D5 by using two surface-bonded arrays with a 15 mm step. During the solicitation, the strains were detected by the FBGs using a SmartScan II interrogator and transferred in real time to the PC. Each second, about 1100 time samples are streamed and processed. When a minimum strain level is exceeded, in order to eliminate background noise, data are processed according to the previously defined logic. The real-time HUMS code takes this data and processes it separately for each SHM code instance in execution. As damage is detected, the index associated with each FBG is printed in the prompt window (Figure 24), and an acoustic signal is also generated and transferred to the sound card, making it audible to the operator.

**Figure 25:**
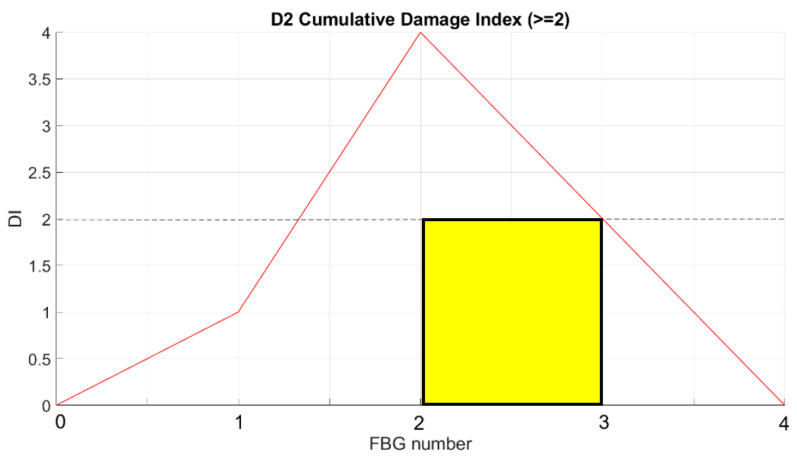
SHM system readout for D2.

**Figure 26:**
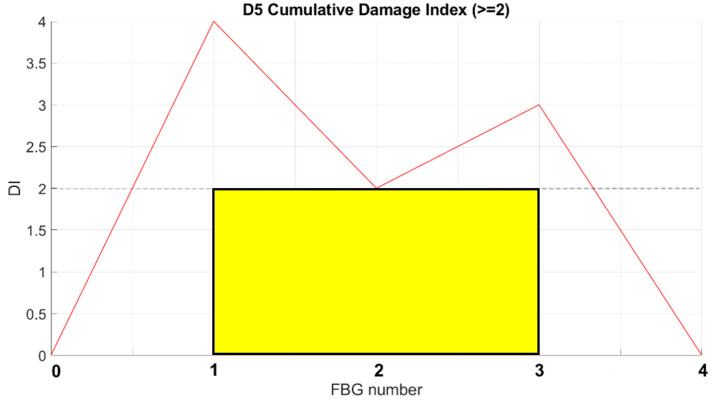
SHM system readout for D5.

In the described setup, as many as three different instances run at the same time. The SHM readouts are provided in Figure 25 and Figure 26, for D2 and D5, respectively. According to a comparison with the sensor layout (Figure 10a), the results are compliant with the array position. The edge of damage D2 to be detected is on left side, and the FBGs on this edge (2 and 3) exceed the threshold level (Figure 25). A comparison with the sensor layout (Figure 10b) shows that the results are compliant with the array position. The edge of damage D5 to be detected is on right side, and the FBGs on this edge (1 and 2) whose exceed the threshold level (Figure 26).

**Figure 27:**
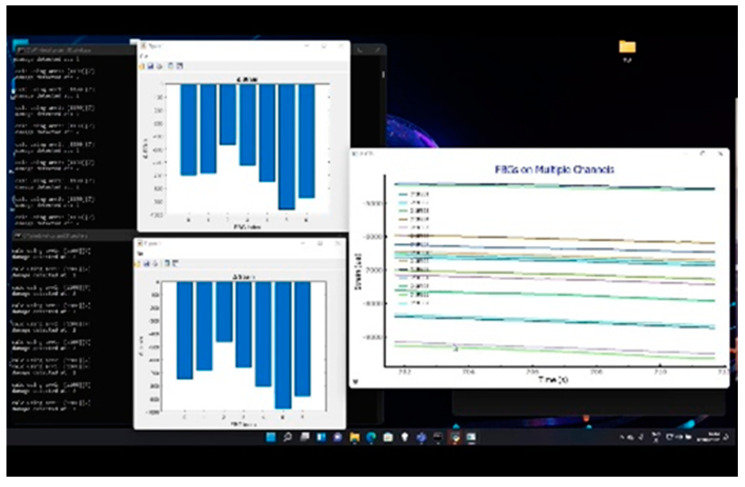
Multiple SHM code instances running simultaneously.

This experimental test allowed us to verify and confirm the correct behavior of the whole system in its full project configuration with multiple SHM executables operating simultaneously on the same data coming from the interrogator. A multiple-instance configuration permits simultaneous execution of the same SHM code on more than one sub-group of FBGs, for instance, to elaborate each fiber separately. Each instance receives and operates on the same data. The Figure 27 depicts a screen capture of the computer’s desktop during the test campaign. The image shows three simultaneous SHM instances: two instances expressing the SHM operation and another instance concentrated on a graphical representation of current strain data to provide the operator with direct information about the structural behavior. The same data are elaborated at each time instant.

A recap of the main results achieved in terms of sensor length detection and presence damage edge detection is provided in Table 1.

## 6. Conclusions

In this paper, we report preliminary experiments carried out on a complex lab test article, i.e., a composite beam, with the aim of testing a real-time SHM system to detect the possible presence of a flaw. The objective fault length was established in the order of tens of mm up to 80 mm, which is representative of the debonding length after which the structural safety factor approaches 1.0.

Two different applications were performed: one implementing a modular FBG sensor network to test and tune the system and another using fixed-step FBG arrays to verify the capability of the developed SHM system.

Data were analyzed and elaborated through different parallel SHM algorithms, their consequent output was translated into audible signals to the operator and the detection points were shown on a graph. A minimum excitation threshold was determined to prompt the system reveal the presence of a flaw. Update intervals can be estimated in the range of a single second.

For the specific test article considered, the system proved its capability in detecting damage, and appreciable results were achieved with respect to the estimation of the damage length. The tested applications confirmed that the proposed SHM system is able to point out the “edge” of a damage region, which is intended as the starting (or ending) point of the occurring fault.

The robustness of edge indication should be improved, as a some oscillation was observed during the load increase. Moreover, the possibility of inserting counters that could make the prediction more robust and reduce the possibility of false positives should also be addressed.

Furthermore, the attained results refer to static or quasistatic conditions, i.e., situations in which the strain field varies slowly with respect to the characteristic acquisition and elaboration times. It is of some interest to investigate the potential behavior of the SHM system in terms of the variation time of the strain field with respect to the operation time of the proposed SHM system (1 Hz). In this regard, it should be considered that a lot depends on the interrogator and its ability to screen data with an adequate frequency. In the current investigation, the upper limit was found to be approximately 1 kHz, which is congruent with the interrogator performance. In such a case, the characteristics of the signal may be captured and elaborated by the SHM system, which can manage those data without any reference to their original time feature. Such elaboration can occur within 1 sec. In particular, the actual bottom limit for the available configuration at a 1250 Hz acquisition is estimated to be approximately 300–400 Hz, representing characteristic variation, which is sufficient to register both the frequency and amplitude of the reported signal. This is important with respect to characteristic frequencies of ordinary structural components, which are typically bounded by the 100 Hz band.

Future research should consider the implementation of larger FBG networks with larger associated computational loads and larger observation regions. Analysis of FBG network density should also be performed, scaling exportability towards more complex systems.

## Figures and Tables

**Table 1:** Main results for damage detection by FBG sensors.

Damage	D3 Length (mm)	D2 Edge Sensors (ID)	D5 Edge Sensors (ID)
*Reference*	*40*	*2*,*3*	*1*,*2*
Estimated	50	2,3	1,2,3

## Data Availability

Data are not available publicly for confidentiality reasons.
